# Pharmacological Evaluation of Withanolide A for Anti‐Alzheimer’s Effects: Inhibition of Mitochondrial Permeability Transition Pore (mPTP)

**DOI:** 10.1002/alz.086073

**Published:** 2025-01-09

**Authors:** Lalit Sharma, Yogesh Gupta

**Affiliations:** ^1^ Shoolini university Solan Himachal Pradesh India, Oachghat‐Kumarhatti Highway Solan India; ^2^ Shoolini University, Solan, Himachal Pradesh India

## Abstract

**Background:**

Alzheimer’s disease (AD) is a complex neurodegenerative disorder characterized by progressive cognitive decline, neuroinflammation, and mitochondrial dysfunction. In Alzheimer’s, abnormal Mitochondrial Permeability Transition Pore (mPTP) activity may contribute to mitochondrial dysfunction and neuronal damage. Withanolide A, a naturally occurring compound derived from *Withania somnifera*, have shown potential neuroprotective effects in various neurological disorders. This study aimed to investigate the anti‐Alzheimer’s effects of Withanolide A and explore their mechanism of action through the inhibition of mPTP.

**Method:**

In our study, we employed both *in vitro* and *in vivo* AD models to assess the impact of Withanolide A on cognitive function, amyloid‐beta (Aβ) pathology, and mitochondrial function. The *in‐vivo* study was carried out on Swiss albino mice. The intracerebroventricular injection of the Aß (1‐42) peptide into the ventricular system of the mouse brain was employed for the induction of the AD. Cognitive function was evaluated through a battery of behavioural tests like Morris water maze, Y maze, and open field test while amyloid‐beta (Aβ) pathology was quantified using immunohistochemical analyses. Mitochondrial function was meticulously characterized through measurements of membrane potential, reactive oxygen species (ROS) production, and adenosine triphosphate (ATP) levels.

**Result:**

Our results demonstrated that Withanolide A significantly improved cognitive performance in AD behavioural mouse models, accompanied by a reduction in Aβ deposition. Importantly, Withanolide A exhibited a potent inhibitory effect on mPTP opening, thereby preserving mitochondrial membrane potential and attenuating ROS production. Further, ATP levels were sustained, indicative of improved mitochondrial bioenergetics. Subsequent exploration of molecular mechanisms elucidated the downregulation of pro‐inflammatory cytokines and upregulation of neurotrophic factors following Withanolide A treatment. These findings suggest a direct link between the anti‐Alzheimer’s effects of Withanolide A and their modulation of mitochondrial function through mPTP inhibition. These results highlight the multifaceted neuroprotective mechanisms employed by Withanolide A in mitigating AD pathology.

**Conclusion:**

Our study provides compelling evidence for the potential of Withanolide A as a therapeutic intervention for AD. The observed inhibition of mPTP, coupled with improvements in cognitive function and mitigation of Aβ pathology, positions Withanolide A as a promising candidates for further research in the pursuit of effective treatments for AD.

